# Impact of Migraine and Its Management in 5 Gulf Countries: Results of a Social Media Platforms Listening Study

**DOI:** 10.2196/82843

**Published:** 2026-10-09

**Authors:** Taoufik Alsaadi, Abubaker Almadani, Suhail Al-Rukn, Alessandro Terruzzi, Ali Mohamed Hassan, Osama Robin El-Alamy, Ali Al Balushi, Fatema Abdullah, Nadine Tarcha, Haytham Mohamed Ahmed, Mohamed Hegab

**Affiliations:** 1 Neurology Department, American Center of Psychiatry & Neurology (ACPN), Delma St. (13th) & Al Karama St (14th) Intersection, BHC Compound No.32, Abu Dhabi, 50000, United Arab Emirates, 971 526028688; 2 Department of Neurology, Rashid Hospital, Dubai, United Arab Emirates; 3 Department of Neurology, Mediclinic City Hospital, Dubai, United Arab Emirates; 4 Department of Neurology, Sheikh Tahnoon Medical City and Tawam Hospital, Al Ain, United Arab Emirates; 5 Department of Neurology, Hamad Medical Corporation, Doha, Qatar; 6 Department of Neurology, Khoula Hospital, Muscat, Oman; 7 Department of Neurology, Salmaniya Medical Complex Governmental Hospitals, Manama, Bahrain; 8 Department of Medical Affairs, Pfizer Inc. LTD, Dubai, United Arab Emirates

**Keywords:** migraine, social media, patient concerns, self-management, search patterns, online conversations, search trends

## Abstract

**Background:**

Migraine is a common neurological disorder that affects a significant proportion of the global population but remains largely underdiagnosed and undertreated. This study analyzed discussions related to migraines in English and Arabic across Kuwait, Bahrain, the United Arab Emirates (UAE), Oman, and Qatar.

**Objective:**

Social media listening may help to identify gaps in migraine management and guide the development of strategies to address them. This study aims to analyze discussions related to migraines in both English and Arabic across various social media platforms. By applying social analytics to the bilingual discourse on migraines in the Gulf region, a region that is often underrepresented, we seek to integrate clinical perspectives with regional digital conversations and identify potential gaps in awareness and care pathways.

**Methods:**

The study was conducted using a retrospective and descriptive approach to social analytics. The study analyzed discussions about migraines from over 100 million websites in English and Arabic across Kuwait, Bahrain, the UAE, Oman, and Qatar between January 1, 2018, and June 30, 2022. Vendor-implemented, rule-based processes, including keyword matching, language identification, and structured data extraction, were used to evaluate publicly available content in English and Arabic.

**Results:**

Out of 53,000 mentions, 33,920 (64%) occurred on X (formerly Twitter), and men were 1.1 times more likely to discuss migraines than women. Among patient conversations, the impact of migraines on overall health was often discussed, including mentions of pregnancy 1060 (2%), vision problems 1325 (2.5%), and sleep disruption 1590 (3%). Medications like paracetamol, aspirin, ibuprofen, and triptans were frequently mentioned, alongside self-management treatments such as cannabidiol softgels, cupping, and aroma oils. Patients often used TikTok to share experiences and seek advice. Many patients actively sought to connect and engage with other migraine sufferers for support and to share experiences. Social media served as a valuable source of information for patients, with health care professionals (HCPs) actively sharing insights and resources through these platforms.

**Conclusions:**

The study finds that analysis of migraine discourse across the 5 Gulf countries reveals active engagement by patients and HCPs in migraine-related discussions on various social media platforms. However, many rely on self-management and alternative medicine instead of seeking professional medical help, which leads to improper management of symptoms. The findings should be regarded as compelling descriptive signals in discourse rather than predictive or inferential outcomes. Social media listening may help to identify possible gaps in management and suggest areas for improvement, but these findings need further validation.

## Introduction

Migraine, a distinct type of headache, manifests as recurring episodes of intense throbbing or pulsating pain that are often localized to one side of the head and persist for 4 to 72 hours [1]. Globally, migraine ranks as the second leading cause of disability and is a common neurological condition characterized by recurrent, lifelong headaches [2]. With over a billion people affected worldwide, the impact extends beyond individual sufferers to their families, friends, colleagues, employers, and others [3]. A review article published in 2023 estimates that global migraine prevalence is approximately 14% to 15% [4]. In terms of health burden, migraines account for 4.9% of the global population’s ill health, measured in years lived with disability [4]. Migraine typically begins at puberty and predominantly affects individuals aged between 35 and 45 years [5].

A study found that the prevalence of migraines in the general population ranged widely from 2.6% to 32% [6]. The prevalence of migraines exhibited notable variations among different Arab countries, despite their sharing similar cultural, economic, ethnic, and climatic backgrounds. For instance, the rates of migraine in Saudi Arabia were higher than those in Oman and Qatar [6-9].

Amid the challenges of living with migraines, social media platforms have emerged as an instant resource for individuals seeking support and information [10]. These platforms might provide immediate access to empathetic peers who understand their experiences, offering validation and reassurance. The term infodemiology combines information and epidemiology and is defined as the study of the distribution and determinants of information in electronic media—predominantly the internet—within populations. The ultimate objective of infodemiology is to inform public health practice and policy [11]. Potential infodemiology indicators include the automated collection and analysis of data reflecting the prevalence, volume, and patterns of information across websites and social media platforms [11]. These metrics may encompass levels of online “chatter” within discussion forums, blogs, and social media platforms such as X (X Corp), as well as user activities captured through search engine queries and related digital interactions [11].

Social media listening is an effective tool for accessing resources on migraine research [12]. The popularity of health-related social media is growing rapidly, as it reflects real-world data [12]. Beyond emotional support, social media may serve as a vital channel for sharing insights into migraine management and exploring treatment options [12]. Patient groups on social media enhance this support system by providing a platform for individuals to openly share their experiences, symptoms, and challenges related to migraines. However, it is essential to understand that social media support should complement, not replace, professional health care advice. Individuals should always consult health care professionals (HCPs) for an accurate diagnosis, personalized treatment plans, and medical guidance.

We conducted a social analytics study to gain insights into the conversation surrounding the understanding and management of migraines across the 5 Gulf countries. These insights will also help raise awareness of available treatment options and assist HCPs in managing the disease more effectively. By applying social analytics to the bilingual (Arabic and English) discourse on migraines in the Gulf region, which is often underrepresented, we aim to integrate clinical perspectives with regional digital conversations and identify potential gaps in awareness and care pathways.

## Methods

### Study Design

#### Overview

The study, commissioned by Pfizer in June 2022, was conducted by Rila Global Consulting using a retrospective, descriptive social analytics approach. Over 100 million websites were crawled, and data spanning up to 4.5 years related to migraines were analyzed. In addition to automated data extraction, analysts experienced in health communication and who were familiar with Gulf Arabic dialects and bilingual (Arabic-English) online discourse conducted a manual review. This review aimed to interpret discussions related to migraines and identify relevant audiences, including individuals and HCPs across the 5 Gulf countries. The study analyzed conversations from January 1, 2018, to June 30, 2022, from Kuwait, Bahrain, the United Arab Emirates (UAE), Oman, and Qatar. Vendor-implemented, rule-based processes, including keyword matching, language identification, and structured data extraction, were used to evaluate publicly available content in both English and Arabic. This process was applied selectively to improve the interpretation of content identified through query-based retrieval. The manual review was not intended to be a formal qualitative annotation exercise and did not adhere to a predefined sampling framework or structured sampling protocol. Dual-independent coding and interrater validation procedures were not implemented, and no formal training protocols for extractors or standardized annotation guidelines were established. As a result, interrater agreement metrics were not calculated. The refined data were processed using automation implemented by the vendor, which included language identification, keyword and query matching, and rule-based parsing. No supervised or unsupervised machine learning or formal natural language processing models were developed, trained, or validated as part of this study. Automated outputs were supplemented by manual expert review to enhance contextual interpretation, especially for Arabic language content. As a result, the findings should be viewed as descriptive and supportive rather than as a validated qualitative classification process.

#### Platform and Source Acquisition

The data collection process involved gathering information in both languages, with a focus on conversations originating from the 5 Gulf region countries. Searches were conducted to gather terminology, abbreviations, and hashtags related to migraines. Data were collected exclusively from publicly accessible digital platforms, including social media networks such as X (formerly Twitter), blogs, online forums, news media outlets, and health information websites. Content originating from the UAE, Kuwait, Bahrain, Oman, and Qatar was included. Resources in both English and Arabic were incorporated. Private accounts, restricted content, or personally identifiable information were not accessed or retained. The dataset reflects publicly available digital discourse and may not be representative of the broader patient population. A total of 53,000 migraine-related mentions were included in the final dataset and underwent automated processing. Manual review was applied across the dataset to support contextual interpretation and classification. Key variables extracted included audience type, topic category, treatment discussions, platform source, and language.

#### Keyword and Hashtag Query Design

Rila Global Consulting developed a structured set of queries designed to capture migraine-related terminology, abbreviations, and hashtags. The key terms and metrics are listed in Table 1. The collected data were then segmented to explore various topics, focusing on specific audiences, including patients, HCPs, health authorities, and medication products. The query library included medical terminology, lay expressions (eg, headache), colloquial language, and hashtags relevant to migraine discourse. The full bilingual query set, including dialectal Arabic, Arabizi, and spelling variants, was proprietary and not retained in a publishable format, limiting reproducibility. Formal sensitivity analyses comparing alternative keyword configurations were not conducted. Therefore, potential keyword-driven capture bias cannot be excluded. Migraine-related treatment discussions were identified descriptively within the dataset based on keyword matches and contextual cues. Influencer identification was conducted separately using descriptive engagement indicators (eg, frequency of posts, likes, shares, and visibility), rather than as part of the query design process. This approach enabled the structured capture of migraine-related discourse for subsequent descriptive analysis.

**Table 1.** Key social media terms and metrics used in the keyword and hashtag query design.

Term	Definition
MENTION	Mentioning the brand, a product, or an account in an online post
AUTHOR	The nickname, username, or full name of the individual or organization who posted a mention
TWEETS	A post on X (formerly Twitter)
RETWEETS	Resharing another account’s tweet to all your followers by clicking on the “retweet” button
FOLLOWING	The number of people the tweeter follows
FOLLOWERS	The number of people following the tweeter
IMPRESSIONS	The combined sum of the followers of the tweet author and the followers of any user that retweeted the post
REACH	An estimate of how many individuals may have seen a piece of content based on its engagement (eg, likes, comments, retweets, replies) and traffic (eg, average site visitors, monthly visitors) that is comparable across data sources and platforms
ENGAGEMENT	The total number of times a user interacts with a tweet or post, as measured by a range of metrics that include likes, comments, replies, shares or retweets, click-throughs, and saves on a post
TRAFFIC	The average number of visitors to a website, mobile app, or profile

#### Deduplication and Bot or Spam Handling

Following data extraction, the dataset underwent a multistage cleaning process. Duplicate posts and syndicated content were first removed to eliminate repeated material. Language filtering was then applied to retain only English and Arabic language content. Geolocation filtering, based on available metadata and platform-specific location indicators, was used to ensure that all entries were relevant to the targeted Gulf Cooperation Council countries. Bot-generated, spam, and other automated content were filtered using vendor-implemented detection tools. Quantitative metrics on exclusion rates, such as the proportion of bots removed, were not retained and therefore cannot be reported. A “mention” may include both original posts and reposted content, depending on platform conventions. No sensitivity analyses excluding high volume or high-reach accounts were conducted, and no weighting by author reach was applied in quantitative summaries. To maintain ethical standards, only publicly accessible posts were included in the analysis. No personal or personally identifiable information was accessed, collected, stored, or analyzed at any stage of the study.

#### Language Identification and Mixed-Language Posts

Refined data were analyzed using vendor-implemented, rule-based language identification and keyword recognition processes, accompanied by expert review to ensure contextual accuracy for Arabic language content. No machine learning models, whether supervised or unsupervised, were trained or validated in this study, nor were any formal natural language processing techniques used.

#### Classification Logic

Posts were categorized according to multiple dimensions, including topic (symptoms, diagnosis, emotional impact, and treatment discussions), audience type (patients, caregivers, HCPs, influencers, or public), and platform characteristics (X, forums, or news websites). Audience categories were defined as follows: patients (self-reported migraine experience), caregivers (discussing the care of a patient with a migraine), HCPs (self-identified HCPs or health care institutions), influencers (high-visibility accounts with substantial engagement), and public (general migraine-related discussions without any personal or professional affiliation). Classification was based on self-identification, linguistic cues, profile information, and platform context.

This categorization was achieved through a combination of query-based segmentation, platform context, and content characteristics, such as source type (individual authors, institutional pages, or media outlets). Categories were not mutually exclusive, and a single post or author could reflect multiple roles or perspectives. No formal annotation codebook, predefined classification schema, or validation framework was developed, as the analysis was designed for descriptive segmentation of online discourse rather than formal audience modeling. Classification was rule-based and descriptive, relying on self-identification, linguistic cues, and platform context, supported by manual expert review. Trained analysts conducted a manual review process to validate the information and ensure consistency. No formal annotation protocol was established, and interrater reliability metrics were not assessed.

Influential authors were identified using descriptive indicators including frequency of migraine-related posts, engagement metrics (likes, comments, shares, and reposts), follower count, visibility, and cross-platform presence. Authors demonstrating substantial reach, sustained engagement, or a notable impact on online discussions were classified as key influencers. No predefined quantitative thresholds were applied, and influencer impact was assessed descriptively without causal or statistical modeling.

#### Descriptive Nature of Analysis

The analytical approach used in this study was descriptive and exploratory in nature. Data were aggregated from publicly available online discussions using query-based retrieval rather than structured sampling. As such, the outputs reflect patterns in digital discourse and are not intended to represent population-level estimates or to support inferential statistical conclusions. All reported percentages were calculated using the total dataset of 53,000 migraine-related mentions as the denominator, unless otherwise specified, representing the proportion of total mentions identified during the study period. Confidence intervals, hypothesis testing, and inferential statistical comparisons were not performed because the study was designed as a descriptive social listening analysis rather than a hypothesis-testing study.

#### Validation

Standardized social analytics metrics were applied to quantify the scale and patterns of migraine-related conversations. These metrics incorporated mentions, impressions, reach, engagement, traffic, and follower-based indicators, as defined in Table 1. These metrics were used for descriptive comparison of conversational patterns across countries and platforms. No hypothesis testing, confidence intervals, subgroup analyses, or effect size calculations were performed. Reported proportions represent observed shares of discourse and should not be interpreted as population-level estimates. As no predictive models were developed, performance metrics such as precision, recall, and *F*_1_-score were not applicable.

#### Framework

The 3-phase structure (prediagnosis, diagnosis, and postdiagnosis) was developed as a conceptual framework to provide an organized overview of the journey of a patient with a migraine and to contextualize social media discourse. The framework is heuristic and was not derived directly from the dataset. Posts were not systematically or algorithmically mapped to phases. No qualitative validation or coding reliability assessment was performed. The framework should not be interpreted as an empirically validated patient pathway.

### Ethical Considerations

This study involved the analysis of publicly available data from online platforms and social media on the topic of migraines in English and Arabic. No personal or identifying information was collected or used, and no human participants were involved. All data were analyzed in aggregate form, and individual users were not identifiable. Therefore, ethics approval was not required for this study.

## Results

### Overview

The results indicate a total of 53,000 mentions during the extraction period, averaging 4000 monthly conversations from 23,000 unique authors. About 33,920 (64%) of these discussions took place on X across both languages. Individuals with migraines primarily engaged with content related to migraine triggers and treatments. Men were slightly more likely than women to discuss migraines, with a 1.1-fold higher engagement rate.

### Exploring English Language Migraine Discourse Among Patients in the Gulf Region

The analysis of English language data (N=53,000) regarding migraine discussions among patients in the Gulf region revealed several concerns, including the potential impact of migraines on overall health. Patients discussed various aspects, including the impact of migraine during pregnancy, with this topic accounting for 1060 (2%) of the mentions. Discussions about vision loss made up 1325 (2.5%) of conversations, migraines in relation to influenza represented over 1060 (2%), concerns about connections to heart attacks accounted for 795 (1.5%), and seizures accounted for 1060 (2%). Sleep disruption due to migraines was a major issue, representing over 1590 (3%) of discussions as depicted in Multimedia Appendix 1. Many sufferers used paracetamol for pain relief and better sleep, showing the severe impact of migraines on daily life.

The analysis also shed light on patients’ self-management strategies for migraine relief, including alternative treatments such as cannabidiol (CB) softgels, cupping, acupuncture, and scalp massages. Patients in the Gulf region generally relied on self-treatment or other sources, underestimating the disease condition; hence, there is a need for greater awareness. Local hospitals actively shared information about treatment options and advancements. Throughout the analysis period, prominent news sites played a role in disseminating information and raising awareness about migraines within the Gulf community.

### Migraine Treatments: Insights From Social Media

Among them, aspirin, ibuprofen, and paracetamol emerged as commonly mentioned options, each gaining attention in social media discussions. Ibuprofen stood out, accounting 1590 of 53,000 (3%), particularly on platforms like Tumblr. Mentions of triptans represented a smaller portion of the discourse, mainly because specific product names dominated the conversation. Metoclopramide, though less frequently discussed, was highlighted by HCPs for its potential benefits in migraine therapy. Nonmedication approaches, such as hot and cold therapy products, gained interest, with certain items like facial masks being mentioned in approximately 530 (1%) of discussions. Unconventional remedies, referred to as a “migraine stick,” were cited as alternative methods for soothing migraine symptoms. These findings showcase the diverse range of treatments explored by migraine sufferers and the insights shared within online communities.

### Revealing the Path to Migraine Solutions: Insights From the Consumer Journey

In examining the customer journey for migraine treatment solutions, social media has emerged as a prominent method for discovering effective remedies. Platforms like TikTok (ByteDance) have become essential for users to share personal experiences and identify potential treatments, often leading them to purchase products on websites. In the Gulf region, nonpharmacological approaches to migraine management, including naturopathy, plant-based diets, and veganism, were present in the dataset and accounted for 2120 (4%) of discussions. Various sources, such as the *Times of Oman* and blogs, described naturopathy and plant-based diets in relation to migraine symptoms; these sources were referenced in patient discussions [13]. Closed groups like the UAE Migraine Facebook group offer a safe space for users, while platforms such as X, Tumblr, and blogs facilitate discussions between patients and health care providers (HCPs). These professionals play a crucial role, offering advice on medication, when to seek help, and strategies for managing migraines.

### HCP Engagement and Thought Leadership in Migraine Management

While webinars accounted for just 1% (530/53,000) of overall mentions, HCPs emerged as prominent participants, actively sharing event details and YouTube (Google) links. Discussions on migraine prevention and acute attack management highlighted the role of regional health care conferences in raising migraine awareness and elevating the Gulf’s visibility in neurology and migraine care [14]. These conferences featured local and international HCPs, amplifying the region’s expertise globally. With a robust online presence, including YouTube and X, conference organizers effectively disseminated migraine-related insights to a wide audience, including patients, HCPs, and the public.

Regional media interviews similarly emphasized that the UAE was preparing for a transformative shift in migraine management, with discussions of migraine prevalence, demographics, and the importance of local data collection [15]. Key trends included higher prevalence among women and a concentration of migraine sufferers within the fertile age range of 20 to 40 years. These insights highlighted the active involvement of HCPs and regional organizations in advancing migraine management practices and raising awareness in the Gulf region.

### Insights From the Gulf

Across 5 Gulf countries, the migraine treatment discourse included contributions from both HCPs and patients. Discussions focused on publication announcements, regulatory updates, and other developments in the field. The conversations extended beyond general discussions about migraines to specific medications and treatments. Gulf-based patients shared and discussed international news, including updates related to US Food and Drug Administration (FDA) approvals and announcements from pharmaceutical companies. These discussions indicate that treatment-related announcements and migraine therapy updates were present in the regional online discourse. A study on oral calcitonin gene–related peptide (CGRP) receptor antagonists for migraines gained attention among Gulf authors and contributed to related discussions [16]. These observations suggest that some social media users in the region discussed such announcements and findings in the study dataset.

### Insights Into Arabic Language Migraine Discourse in the Gulf Region

Arabic-speaking patients in the Gulf region gather information about migraines primarily from social media, news, and television. Social media platforms serve as channels for sharing experiences and seeking advice, including viral tweets that invite audience participation in TV discussions featuring medical experts. Other information sources include hospital-led webinar announcements about updates in migraine management [17]. There were also discussions suggesting that migraine symptoms were caused by subvariants of the coronavirus. FDA-approved options for migraine prevention, including medical devices, mobile apps, and innovations in the field, have been acknowledged by patients with migraines in the Gulf [18].

Government involvement and public health concerns are also prevalent in the discourse, with patients in Kuwait advocating for improved access to migraine medication and patients in Bahrain calling for measures to address environmental triggers such as noise pollution. Discussions surrounding the effectiveness and side effects of painkillers, particularly paracetamol and stronger medications like sumatriptan and diclofenac, also shed light on the challenges faced by patients with migraines in managing their symptoms.

### Navigating the Journey of Patients With Migraines

To provide a principal structure for interpreting discourse patterns, the findings were organized into 3 conceptual stages of the migraine journey: prediagnosis, diagnosis, and postdiagnosis, as shown in Figure 1.

**Figure 1.**
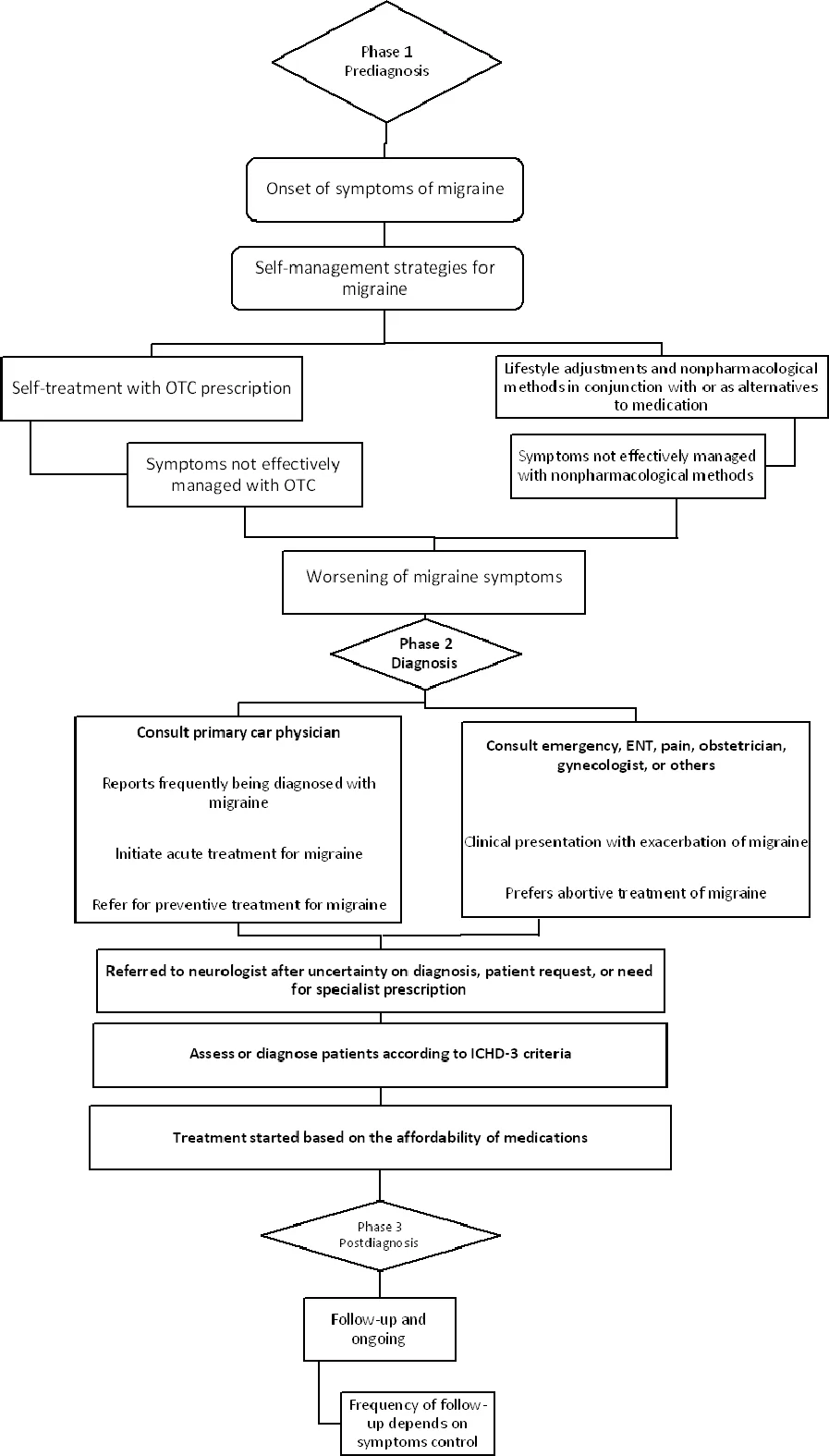
Navigating the journey of patients with migraines. ENT: ear, nose, and throat (specialists); ICHD-3: International Classification of Headache Disorders, Third Edition; OTC: over the counter.

#### Phase 1: Prediagnosis

The journey of a patient with a migraine often begins with the onset of symptoms, which can include severe headaches, nausea, sensitivity to light and sound, and aura. Initially, many individuals attempt to manage these symptoms through self-treatment with over-the-counter medications such as ibuprofen or acetaminophen [19]. Some patients explore nonpharmacological options like lifestyle changes, dietary adjustments, stress management techniques, and other approaches. While these methods can be beneficial for some, they may not always provide sufficient relief, highlighting the need for professional health care guidance.

#### Phase 2: Diagnosis

At this point, patients typically consult a primary care physician. These physicians frequently diagnose migraines and initiate acute treatment to address the immediate symptoms. In cases where migraine symptoms worsen, patients may consult emergency services, ENT (ear, nose, and throat) specialists, pain management experts, obstetricians, gynecologists, or other relevant HCPs, as applicable. During these consultations, patients may express a preference for abortive treatments to stop migraine attacks. These physicians may refer patients for preventive treatment to manage long-term migraine occurrences. If there is uncertainty in the diagnosis, if the patient requests it, or if a specialist prescription is needed, the primary care physician refers the patient to a neurologist [20]. The neurologist performs a thorough assessment and makes a diagnosis according to the International Classification of Headache Disorders, Third Edition criteria, regardless of any prior diagnosis. Treatment plans are then tailored based on the patient’s affordability and access to medications.

#### Phase 3: Postdiagnosis

Following the diagnosis, ongoing care and follow-up are crucial [21]. The frequency of follow-up appointments is determined by how well the patient’s symptoms are controlled. Alongside medical treatments, lifestyle adjustments and nonpharmacological methods, such as dietary changes, stress management techniques, and regular physical activity, are recommended. This structured approach ensures that patients receive comprehensive care from the onset of symptoms through to effective long-term management, addressing both the immediate and chronic aspects of migraines. These stages do not represent a strictly linear or validated sequence, as migraine care is often recurrent and experiences may overlap.

## Discussion

### Principal Results

The study analyzed migraine conversations from January 1, 2018, to June 30, 2022, focusing on the 5 Gulf countries: Kuwait, Bahrain, the UAE, Oman, and Qatar. Out of 53,000 mentions, 33,920 (64%) were on X, and men were 1.1 times more likely to discuss migraines than women. Although migraines affect both sexes, it is often stereotyped as a condition that primarily impacts women due to its higher prevalence among women and the increased health care resources used by women for migraine management [22].

Conversations among patients in the Gulf region, conducted in English, discussed the impact of migraines on overall health. Patients discussed various aspects, including the impact of migraines during pregnancy, with this topic accounting for 1060 (2%) of the mentions. During pregnancy, migraine episodes can impact a woman’s well-being and raise important considerations for the health of both the mother and baby [23,24]. Conversations related to migraines in relation to influenza represented over 1060 (2%) of mentions; the effect on vision, 1325 (2.5%); concerns about connections to heart attacks accounted for 795 (1.5%); seizures, 1060 (2%); and disruption of sleep caused by migraines, 1590 (3%). Migraines are linked to a broad spectrum of comorbidities, spanning from stress and sleep disturbances to suicide [25].

Therapies mentioned in social media discourse ranged from evidence-based pharmacological treatments to complementary or self-management remedies. Medications such as paracetamol, aspirin, ibuprofen, and triptans were frequently mentioned, along with self-management treatments like CBD softgels, cupping, and aromatherapy oils. Evidence-based therapies for migraines also include newer CGRP-targeting agents in social media discourse. For example, an oral CGRP receptor antagonist has demonstrated efficacy in relieving migraine symptoms compared with a placebo in the acute treatment of migraine attacks in randomized controlled trials [16]. In contrast, social media discussions frequently referenced nonpharmacological or complementary approaches such as acupuncture, cupping therapy, aromatherapy products, and topical remedies. Some nonpharmacological approaches, including behavioral therapies, lifestyle modifications, and certain complementary practices, have supportive but variable levels of evidence. However, other interventions discussed online, such as CBD products or topical remedies, currently have limited or uncertain clinical evidence for migraine management. The widespread discussion of these therapies in online communities may reflect interest in alternative symptom relief. While pharmacological treatments remain the primary approach for managing migraines, concerns over adverse effects and inefficacy have led patients to explore Complementary and Alternative Medicine therapies. More people are looking for these therapies to treat and prevent migraine attacks because they are seen as safe and possibly effective [26].

Online communities on social media serve as hubs for sharing insights, seeking advice, and exchanging experiences related to migraine management [10]. Patients use TikTok to share their experiences and seek advice. Many individuals also join closed groups, such as the UAE Migraine Facebook group, to connect with other migraine sufferers for support and to share their experiences. HCPs and patients are actively engaged in discussions about migraines. A key limitation of social media–derived insights is the influence of the digital divide and related health equity issues. For example, younger individuals generally engage more with social media than older adults, which may result in findings that disproportionately reflect the perspectives of more digitally active users rather than the broader patient population [27].

Migraines impose significant personal and social burdens, affecting not just the individual but also their family. It can disrupt interpersonal relationships and psychological health, jeopardizing the financial stability of the entire family unit [28]. Key themes in English language discourse included concerns about migraines’ impact on health, correlations with other illnesses, and self-management strategies. Patients expressed interest in alternative treatments, while hospitals and news sources disseminated information on treatment options and health care advancements.

Arabic language discourse mirrored similar themes, with a focus on social media as a source of information. Discussions encompassed a wide range of topics, including concerns about migraines’ impact on health, correlations with other illnesses, self-management strategies, medications used by patients to treat migraines, alternative therapies, and the journey of patients with migraines. Patients advocated for improved access to medication and government intervention to address environmental triggers. Various platforms, including scientific journals, news media, and specialized medical websites, played a role in disseminating treatment advancements. The study offers valuable insights into the varied landscape of migraine discussions across the 5 Gulf countries, emphasizing the complex nature of patient experiences, treatment options, and engagement with HCPs.

#### Implications

Social media platforms have become essential tools in health care, facilitating the spread of health information, medical research, education, patient engagement, and professional development [29]. The implications of social media listening in migraines are significant for understanding patient experiences, unmet needs, and communication gaps in real-world settings. By analyzing patient discussions on social media platforms, health care stakeholders can gain insights into symptom burden, treatment perceptions, and common misconceptions surrounding migraine management [12]. These insights may help identify areas where patient education, awareness, and clinical communication require strengthening. Social media listening can also highlight emerging concerns related to treatment effectiveness, adverse effects, and barriers to accessing care that may not always be captured in traditional clinical studies [30]. However, findings derived from social media should be interpreted cautiously, as the data are observational, may not represent the entire patient population, and cannot establish causal relationships. When used responsibly, social media listening can complement traditional research approaches and contribute to more patient-centered strategies for migraine awareness, education, and management. Although social media listening cannot establish prevalence or clinical effectiveness, these patterns may serve as hypothesis-generating signals that can be further validated through epidemiological and clinical research.

#### Recommendations

##### Evidence-Based Recommendations

Emphasis should be placed on accurate diagnosis, differentiation from other headache disorders, and early identification of patients who require preventive therapy or specialist referral. Health care systems should also reinforce primary care to support early recognition and establish clear referral pathways for refractory or atypical cases [31]. Migraine management requires a patient-centric approach that emphasizes structured education, appropriate reassurance, and the establishment of realistic therapeutic goals. Treatment should be individualized according to patient symptoms, triggers, and preferences, with routine monitoring supported by headache diaries. Long-term management should remain in primary care, with stable patients maintained on effective therapy and monitored for any changes [32].

##### Social Discourse–Derived Recommendations

Analysis of patient discussions on social media provides practical, hypothesis-generating signals about symptom burden, perceived treatment effectiveness, adverse effects, self-medication practices, misconceptions, and barriers to timely care that may not be fully captured in conventional studies [30]. Based on these discourse themes, the following actions are recommended: patient education should explicitly address migraines as a neurological disorder rather than a routine headache, explain when recurrent or disabling headache warrants medical assessment, discourage inappropriate reliance on unsupervised analgesic use, clarify the role and expected timeline of preventive therapy, and promote headache diaries for documenting symptoms, triggers, medication use, and treatment response. Clinicians should routinely ask patients about information obtained from social media, correct misconceptions during counseling, screen for medication overuse and adverse-effect concerns, individualize acute and preventive treatment according to patient-reported triggers and preferences, set realistic expectations for response, and refer patients with refractory, atypical, or disabling migraines to specialist care. Policymakers and health systems should convert recurrent public misconceptions into targeted awareness messages, improve access to reliable patient educational materials, support primary care training on migraine recognition and referral criteria, strengthen referral pathways to specialized headache services, and use repeated social listening analyses to identify emerging gaps after guideline updates, therapeutic approvals, or public campaigns. These findings remain nonrepresentative and hypothesis-generating and should not be considered a substitute for clinical evidence; however, when applied judiciously, they can support more specific, patient-centered migraine education, counseling, and service planning. Recommendations derived from social discourse are summarized in Table 2.

**Table 2.** Recommendations derived from social discourse.

Area of value	Insights derived from social media platforms	Recommendations in migraine care
Unmet patient needs	Patients often share pain burden, side effects, access challenges, stigma, and daily-life disruption due to migraines on social media.	Clinicians should routinely ask about pain burden, side effects, stigma, work disruption, and access problems, and then use these answers to tailor counseling, medication plans, and support referrals.
Patient education	Common questions, misconceptions, and recurring concerns appear repeatedly in online discussions.	Use recurring questions and misconceptions to create plain-language patient education on when migraines need medical review, the difference between acute and preventive treatment, safe medication use, trigger management, and when to seek specialist care.
Patient-centered care	Posts frequently illustrate how migraines impact various aspects of life, including work, family relationships, sleep, emotions, and social activities.	Care plans should include discussion of sleep, work, family responsibilities, mood, and social participation, with agreed self-management goals and referral to psychological or occupational support when needed.
Treatment barriers	Patients discuss cost, delayed referrals, medication availability, and confusion about acute vs preventive therapy.	Clinicians should ask directly about cost, access delays, medication availability, and uncertainty about treatment type. Services should provide referral checklists, treatment-navigation guidance, and practical access support.
Monitor misinformation	Unverified remedies and nonevidence-based claims can spread quickly across online platforms.	Clinicians and patient organizations should respond with evidence-based myth-busting messages, clarify risks of unverified remedies, and direct patients to trusted migraine resources.
Service improvement	Online conversations reveal gaps in follow-up, communication, appointment access, and support tools.	Policymakers and service leads should use reported gaps to improve appointment access, follow-up systems, referral pathways, digital support tools, and implementation of migraine-care guidance.
Stigma and emotional burden	Patients may openly share feelings of being dismissed, misunderstood, or isolated.	Clinicians should validate patients’ experiences, screen for anxiety or depression when distress is expressed, offer peer-support options, and use stigma-sensitive language in counseling and aids.

### Future Research Directions

Social media listening studies, combined with structured surveys, patient interviews, or registry data, may help strengthen findings in future in migraine research. Evaluating how themes evolve over time, particularly following guideline updates, therapeutic approvals, or public awareness campaigns, can be beneficial. Future research should account for differences in social media penetration and usage across countries and subgroups (age, sex, and urbanicity) to improve representativeness. Researchers should examine geographic variation in discourse to identify region-specific educational needs or systemic barriers related to migraines. Researchers should design and prospectively assess digital education campaigns to determine whether addressing identified myths or gaps measurably alters knowledge or adherence in migraine management.

### Limitations

One notable limitation of our study is the absence of data from Facebook and Instagram (Meta Platforms, Inc), as these platforms do not permit data crawling. This restriction significantly affects the overall thoroughness of our analysis, especially given the importance of these platforms, where data collection is constrained. This may introduce substantial selection bias due to differing user demographics, privacy settings, and communication norms across platforms. The dataset may overrepresent publicly visible discussions on platforms such as X and the open web while underrepresenting conversations on private platforms.

Therefore, the observed finding that men were 1.1 times more likely to discuss migraines should be interpreted cautiously. Established epidemiological evidence demonstrates a higher migraine burden among women [4,22], suggesting that the gender pattern observed in our dataset may reflect platform-specific engagement behaviors rather than true disease prevalence. Public platforms such as X may overrepresent certain demographic groups, while migraine-related discussions among women may occur more frequently within private or closed communities that were not captured in this analysis. Social media mentions should not be equated with prevalence, and the gender distribution reported here should be interpreted as discussion behavior within a particular platform rather than epidemiological representation. Future research should triangulate social media listening findings with regional survey or epidemiological data to better assess the extent of platform-driven bias.

Formal sensitivity analyses comparing broader versus narrower keyword configurations were not conducted during the original analysis window. As a result, the robustness of keyword expansion strategies could not be quantitatively evaluated retrospectively, introducing the possibility of keyword-driven capture bias; therefore, findings should be interpreted with this limitation in mind. The full bilingual search strategy, including Arabic dialects, spelling variants, and transliterations such as Arabizi, was not systematically retained, nor was code-switching (English and Arabic in the same post) formally addressed, which may limit reproducibility. The complete proprietary query library developed by Rila Global Consulting was not retained in a publishable format, further limiting transparency and reproducibility.

Key statistical elements, including confidence intervals, subgroup comparisons, effect sizes, and corrections for multiple testing, were not performed. Confidence intervals for key proportions and for comparisons by country, language, platform, time period, and audience segment were not included in this analysis. Adjustments for multiple comparisons, such as Bonferroni or false discovery rate corrections, were not performed. Safeguards to ensure analytic independence (eg, a prespecified protocol, independent validation, and data transparency) were not implemented. These factors may affect the robustness of findings and introduce potential bias, particularly in the interpretation of medication-related discourse.

The dataset was collected through extensive web crawling and aggregation of online discussions based on specific queries, rather than through a structured sampling design. As a result, no hypothesis testing, confidence intervals, subgroup comparisons, or effect size calculations were performed. Consequently, the quantitative outputs reflect the proportions of online mentions and engagement in the dataset and should not be interpreted as epidemiological estimates or population-level measures. The findings are descriptive and intended to generate hypotheses rather than provide comprehensive or population-based estimates. The lack of standardized annotation protocols, interrater reliability assessment, and formal classification validation may introduce variability in categorization. This variability should be taken into account when interpreting the results.

Another limitation of this study is that the findings are presented as cumulative data, without temporal trend analysis. The study period (2018 to mid-2022) spans substantial shifts in social media platform usage and online health information–seeking behaviors, particularly during the COVID-19 pandemic. The absence of time-stratified analyses limits the study’s ability to evaluate how discourse patterns, engagement levels, and content characteristics may have evolved over the pandemic period.

The journey of patients with migraines in the “Results” section is a conceptual structure created by the authors to organize themes in the data. It was not derived from a temporal analysis of posts. It should not be interpreted as a validated or strictly linear patient journey, as migraine management is often cyclical, and posts may reflect overlapping or repeated stages of experience.

### Comparison With Prior Work

A 2024 study focused on the unmet needs of patients living with migraines in the Gulf Cooperation Council countries. The findings suggest that the burden of migraines and its impact on patients in these countries are often underestimated and that both diagnosis and treatment are suboptimal [33].

Another study reported that the prevalence of migraines in the general Arab population ranges from 2.6% to 32%. Among medical university students, the estimated prevalence ranges between 12.2% and 27.9%, while among schoolchildren aged 6 to 18 years, it ranges between 7.1% and 13.7%. The study also found that women were more likely to experience migraines than men [6]. In contrast, our findings indicated that men were 1.1 times more likely to discuss migraines on social media than women. This discrepancy may reflect the limitations of social media–derived interpretations, including aspects of the digital divide and health equity wherein participation patterns differ across genders. For instance, women may be more likely to engage in private or closed online groups, which were not captured in our study.

In 2023, a study found that X was the most-used social media platform in Japan, Germany, and France [12]. Our study also revealed that 64% (33,920/53,000) of the discussions took place on X, in both English and Arabic.

On the other hand, a 2019 study found that Facebook emerged as the preferred social media platform for discussions related to migraines, especially within closed, migraine-specific groups [10]. These groups played a crucial role in providing individuals with valuable information and social support, helping them understand and manage their condition effectively. Our study found that closed groups, such as the UAE Migraine Facebook group, offer safe spaces, while X, Tumblr, and blogs facilitate discussions between patients and HCPs. Patients engaged in discussions concerning migraine triggers, treatment options, alternative therapies, and self-management strategies, while HCPs shared insights on medications and medical advancements.

### Conclusions

In conclusion, the analysis of migraine discourse among the 5 Gulf countries highlights the engagement of patients and HCPs in discussions concerning migraines on different social media channels. It is evident that patients in the Gulf region use social media as a primary channel for sharing experiences, seeking advice, and raising awareness about migraines. The involvement of HCPs enriches these discussions by providing expert guidance and participating in educational initiatives. The discourse explores concerns about migraines’ impact on health, correlations with other illnesses, self-management strategies, medications used by patients to treat migraine, and alternative therapies. This helps us understand the diverse perspectives and preferences within the Gulf migraine community.

Many patients turn to self-management and alternative medicine, which can lead to less effective outcomes. This choice to avoid professional medical help may result in inadequate symptom management, highlighting the need for expert health care. To improve migraine care practices in the region, it is essential to bridge this gap by increasing awareness and providing education. These insights will also help raise awareness of available treatment options and dispel misconceptions. Therefore, social media listening may help identify gaps in migraine management and better understand migraine-related concerns, although further validation is required through additional research.

## Supplementary material

10.2196/82843 Multimedia Appendix 1 English language discourse on migraine-related comorbidities among patients in the Gulf region (N=53,000).

## Abbreviation

CBD: cannabidiol
CGRP: calcitonin gene–related peptide
ENT: ear, nose, and throat
FDA: US Food and Drug Administration
HCP: health care professional
UAE: United Arab Emirates
